# A Computationally Efficient Viscoelastic Eukaryotic Cell Model

**DOI:** 10.1007/s10439-025-03772-5

**Published:** 2025-06-19

**Authors:** Pietro Miotti, Matteo Scarpone, Chwee Teck Lim, Igor V. Pivkin

**Affiliations:** 1 Institute of Computing, Faculty of Informatics, Università della Svizzera italiana, Lugano, Switzerland; 2 Swiss Institute of Bioinformatics, Lausanne, Switzerland; 3 Institute for Health Innovation and Technology (iHealthtech), National University of Singapore, Singapore, Singapore; 4 Department of Biomedical Engineering, National University of Singapore, Singapore, Singapore; 5 Mechanobiology Institute, National University of Singapore, Singapore, Singapore

**Keywords:** Eukaryotic cell model, Coarse-grained particle-based method, Dissipative particle dynamics, Viscoelastic model

## Abstract

**Purpose:**

Modeling eukaryotic cell flow in microfluidic devices and capillary networks can be instrumental in assessing how cell mechanics influence its behavior. Due to the viscoelastic characteristics of cells and their capacity for substantial deformation, models that are both detailed and computationally efficient are necessary to explore cell rheology. We present a coarse-grained model for simulating the mechanics of eukaryotic cells in flow, with a focus on the modeling of cell membrane, nucleus, and cytoskeleton.

**Methods:**

The cell and nucleus membranes are represented using surface triangulation, capturing both viscous and elastic properties of the membranes. To maintain computational efficiency while retaining the ability to reproduce the viscoelastic behavior of the entire cell, the complexity of the cytoskeleton model is reduced through the use of the viscoelastic bonds. Dissipative Particle Dynamics is employed to facilitate flow simulations; however, the model is suitable for use in many existing continuum and particle-based methods.

**Results:**

The cell model is calibrated and validated using experimental data from micropipette aspiration and microfluidic experiments involving breast epithelial cells (MCF-10A).

**Conclusion:**

We believe the balance between simplicity and accuracy makes the proposed model a valuable tool for simulating eukaryotic cell mechanics in flow, enabling faster simulations, while also simplifying the parameterization procedure.

**Supplementary Information:**

The online version of this article contains supplementary material available 10.1007/s10439-025-03772-5.

## Introduction

In recent decades, there has been significant growth in research on cell mechanics, a field that explores the mechanical behavior of cells and its implications for diseases such as cancer [1]. Cells possess remarkable mechanical properties, and understanding how forces and mechanical cues influence their behavior is crucial in various aspects of biology and medicine. Experimental techniques like micropipette aspiration, atomic force microscopy, fluidic force microscopy, and microfluidics enable the measurement of cellular forces and estimating mechanical properties [2–5]. In silico models have also been developed for studying and understanding the mechanical behavior of cells and their interactions within microenvironment, providing insights for improving the diagnosis, treatment, and prevention of diseases [6–14].

Mature human red blood cell (RBC) is among the simplest cells to model, as it lacks a nucleus and internal cytoskeleton [15]. Continuum- and molecular-based numerical models have been developed and applied to RBC simulations, varying in the level of detail used to describe the RBC membrane. Continuum models face challenges in accounting for the detailed structure of the RBC, while for atomistic methods it is currently not feasible to simulate an entire membrane, including lipids and membrane proteins. To bridge this gap, coarse-grained mesoscale particle-based methods have been developed in recent years. The first 3D spectrin-based RBC membrane model, introduced by Discher et al. [16], was used to investigate RBC cytoskeleton deformation during micropipette aspiration. This work was later extended by Li et al. [17], leading to a spectrin-level RBC model that became the foundation for further developments. A systematic coarse-graining procedure was proposed for this model, reducing its degrees of freedom by more than two orders of magnitude [18], enabling simulations of RBCs in various types of flow [19–26]. Another widely used approach simulates fluid with the Lattice–Boltzmann method (LB), RBC membrane forces with the finite element method, and RBC–fluid interactions using the immersed boundary method (IB) [27–30]. This approach has been applied to study RBC tumbling-to-tank-trading transitions in shear flow [28], RBC distribution in small bifurcations [29], and large-scale RBC simulations [30]. Other models rely on the Finite Volume method [31], moving particle semi-implicit method [32], coarse-grained Molecular Dynamics [33, 34], and the Stochastic Rotation Dynamic method [35]. Review of these models can be found in [36–39] and references therein.

Other cells found in blood, such as white blood cells (WBCs), platelets, and circulating tumor cells (CTCs), contain internal organelles, including a nucleus and internal cytoskeleton, in addition to the cell membrane. Models of these suspended cells in flow can be categorized into two groups. The first group does not explicitly describe the internal organelles, instead adapting the RBC membrane model with adjusted parameters. For example, the spectrin-based RBC model was extended to represent CTCs and used to study their separation from whole blood samples in a microfluidic device utilizing the deterministic lateral displacement effect [40]. Other models based on the LB-IB method have been applied to CTC membrane deformations [41] and deformable platelet adhesion [42]. The second group of models incorporates internal organelles, such as the nucleus and internal cytoskeleton. Examples include a high-resolution cancer cell model developed to investigate the effects of interacting shock waves [43] and a detailed platelet model used to simulate the dynamic properties of flowing platelets [44]. Review of these models can be found in [45–48], and references therein. Most existing models from the two groups discussed above are not well suited for numerical investigations of cell behavior in flow. The first group of models oversimplifies the cell and fails to accurately describe its behavior under large deformations, while the computational complexity of other models often makes them impractical for applications involving large numbers of cells in flow.

In this work, we propose a model for eukaryotic cells that explicitly implements the membrane, nucleus, and cytoskeleton, which are assumed to be the principal components affecting the cell’s mechanical properties [1]. The model is a simplified version of the one introduced by Lykov et al. [7], obtained by replacing the gel-like structure of the cytoskeleton with viscoelastic bonds. This reduces both the computational complexity of the model and the number of parameters involved. At the same time, the model is still able to capture the viscoelastic nature of the eukaryotic cell. We calibrate and validate the model using experimental data published in [7], which include micropipette aspiration and microfluidics data for the breast epithelial cells (MCF-10A cell line). We note that a similar model has been recently developed by Balogh et al. [49], which also emphasizes the importance of modeling the nucleus in simulations of eukaryotic cells, but it does not explicitly describe the viscous properties of the cell, which are known to play a key role in microfluidic experiments [7, 19, 50].

## Materials and Methods

### Dissipative Particle Dynamics

The simulation method employed in our studies is the Dissipative Particle Dynamics (DPD) [51–53], which we briefly summarize below.

In DPD, the system of interest is modeled by a collection of *N* particles, with *i*-th particle coordinate and velocity being ${\textbf {r}}_i$ and ${\textbf {v}}_i$, respectively. We assume that all particles have equal mass *m* (we set $m=1$). The time evolution of position and velocity of each particle is then described by Newton’s equations of motion,

1 $$\begin{aligned} \frac{d{\textbf {r}}_i}{dt}={\textbf {v}}_i \end{aligned}$$

and

2 $$\begin{aligned} \frac{d{\textbf {v}}_i}{dt}={\textbf {f}}_i \end{aligned},$$

where ${\textbf {f}}_i$ is given by the contribution of three different forces,

3 $$\begin{aligned} {\textbf {f}}_i = \sum \limits _{i \ne j} \left( {\textbf {f}}^C_{ij} + {\textbf {f}}^D_{ij} + {\textbf {f}}^R_{ij} \right) . \end{aligned}$$

A soft conservative repulsive force, $\textbf{f}^C_{ij}$, is defined as

4 $$\begin{aligned} \textbf{f}^C_{ij} = {\left\{ \begin{array}{ll} a_{ij}(1-r_{ij}/r_c) \hat{{\textbf {r}}}_{ij},& r_{ij} < r_c \\ 0,& r_{ij} \ge r_c, \end{array}\right. } \end{aligned}$$

where parameter $a_{ij}$ defines the force magnitude for interacting particles *i* and *j*, ${\textbf {r}}_{ij}={\textbf {r}}_i-{\textbf {r}}_j$, $r_{ij} =|{\textbf {r}}_{ij}|$, $\hat{{\textbf {r}}}_{ij}={\textbf {r}}_{ij}/|{\textbf {r}}_{ij}|$, and $r_c$ is the cutoff radius beyond which interactions between particles are neglected. A dissipative force, ${\textbf {f}}^D_{ij}$, is given by

5 $$\begin{aligned} {\textbf {f}}^D_{ij}=-\gamma ^D w^D(r_{ij}) ({\textbf {r}}_{ij} \cdot {\textbf {v}}_{ij}) \hat{{\textbf {r}}}_{ij}, \end{aligned}$$

where its magnitude is controlled by the dissipative force coefficient $\gamma ^D$, $w^D(r_{ij})$ is a weighting function, and ${\textbf {v}}_{ij}={\textbf {v}}_i - {\textbf {v}}_j$. A random force, ${\textbf {f}}^R_{ij}$, is defined as

6 $$\begin{aligned} \textbf{f}^R_{ij} = \sigma w^R(r_{ij})\theta _{ij} \Delta t^{-1/2}\hat{{\textbf {r}}}_{ij} , \end{aligned}$$

where its magnitude is controlled by the random force coefficient $\sigma$, $w^R(r_{ij})$ is a weighting function, $\theta _{ij}$ is a the gaussian random noise with zero mean and unit variance, and $\Delta t$ is the time step in the modified velocity-Verlet algorithm used to integrate equations of motion [52]. From the fluctuation–dissipation theorem, relations between coefficients $\gamma ^D$, $\sigma$, and functions $w^D(r_{ij})$ and $w^R(r_{ij})$ are derived as

7 $$\begin{aligned} \sigma ^2=2\gamma ^D k_BT, \end{aligned}$$

8 $$\begin{aligned} w^D(r) = [w^R(r)]^2, \end{aligned}$$

with temperature, *T*, and Boltzmann constant, $k_B$ [54]. We use a generalized weighting function $w^R(r) = (1-r/r_c)^s$ with parameter $s = 0.75$ [55]. The DPD parameters used in simulations are taken from Reference [7].

### Cell Model

**Fig. 1:**
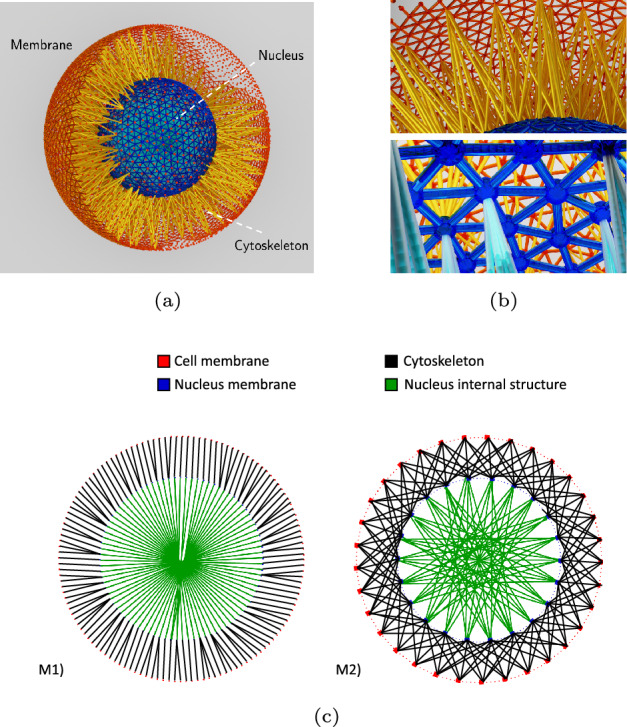
Eukaryotic cell model. **a** Visualization of the entire cell model with its main components. **b** Detailed visualization of the cytoskeleton bond topology (above) and the nucleus membrane with cell and internal cytoskeleton bonds (below). (c) 2D sketch illustrating different topologies used for cytoskeleton model construction with the same bond density $\rho = 1$. In Model 1 (left), eligible cell outer membrane particles are linked directly with the nearest eligible particles in the nucleus membrane, while nucleus membrane particles are linked to the most distant nucleus membrane particles. In Model 2 (right), each selected outer membrane particle is connected to the *w* closest nucleus membrane particles, and similarly each selected nucleus membrane particle is connected to the *w* most distant nucleus membrane particles.

The computational model of the cell is composed of three main components: an outer membrane, a nucleus, and a cytoskeleton complex that is placed between the outer membrane and the nucleus (Fig. 1a, b).

#### Membrane Model

The cell and nucleus membranes are each represented by a mesh composed of $N_v$ vertices, $N_e$ edges (bonds), and $N_t$ triangles [16–18]. Each vertex is modeled by a DPD particle with position ${\textbf {r}}_i$ and velocity ${\textbf {v}}_i$, while edges connect pairs of particles and form membrane surface triangulation. The elastic properties of the membrane are described by the sum of the contributions of the in-plane shear energy, the bending energy, and constraints of surface area and volume conservation. The in-plane shear energy $U_s$ is defined as

9 $$\begin{aligned} & U_s = \sum \limits _{i \in bonds}\bigg [\frac{k_BTl_{max}}{4p} \frac{3x_i^2-2x^3_i}{1-x_i}\bigg ] \nonumber \\ & + \sum \limits _{\alpha \in triangles} \frac{1}{A_\alpha }\bigg [ \frac{3\sqrt{3}k_BTl^3_{max}x^4_0}{64p}\frac{4x^2_0-9x_0+6}{(1-x^2_0)} \bigg ], \end{aligned}$$

where $A_\alpha$ is the area of triangle $\alpha$ defined by three vertices, $x_i=l_i/l_{max}$, $x_0=l_0/l_{max}$, $l_i$ is the length of bond *i*, $l_0$ is the bond equilibrium length, $l_{max}$ is the maximum bond extension, and *p* is the persistence length. The in-plane energy consists of two components, each represented by a summation: the entropic energy stored in spectrin protein links and the elastic energy stored in the lipid membrane and other protein structures. This formulation was originally introduced for modeling the erythrocyte membrane by Discher et al. [16] and later extended in subsequent works [17, 18]. The membrane bending resistance is defined by

10 $$\begin{aligned} U_b=\sum \limits _{\alpha ,\beta \ pair}k_b[1-cos(\theta _{\alpha \beta }-\theta _0)], \end{aligned}$$

where $k_b$ is the bending modulus, $\theta _{\alpha \beta }$ is the angle between two adjacent triangles having a common edge, and $\theta _0$ is the equilibrium angle. For area and volume conservation, two additional terms are included in the model:

11 $$\begin{aligned} U_a=\frac{k_a k_BT(A-A_0)^2}{2l_0^2A_0}, \quad U_v = \frac{k_v k_BT(V-V_0)^2}{2l_0^3V_0}, \end{aligned}$$

where $k_a$ and $k_v$ are area and volume constraint coefficients, $A_0$ and $V_0$ are membrane equilibrium area and volume, and *A* and *V* are the current area and volume. To model the membrane viscosity, a dissipative force, ${\textbf {f}}^D_{ij}$, is added to the particles of each bond in the membrane,

12 $$\begin{aligned} {\textbf {f}}^D_{ij} = -\gamma ^T {\textbf {v}}_{ij} -\gamma ^C ({\textbf {v}}_{ij} \cdot \hat{{\textbf {r}}}_{ij})\hat{{\textbf {r}}}_{ij}, \end{aligned}$$

where $\gamma ^T$ and $\gamma ^C$ are the coefficients for the translational and rotational components of the dissipative force [56, 57]. To reduce the number of parameters in the model, we assume these coefficients to be equal $\gamma ^T = \gamma ^C = \gamma$, where $\gamma$ is the parameter controlling the viscous contribution of the membrane. In addition to the dissipative force, a random force, ${\textbf {f}}^R_{ij}$, is added to the particles of each bond in the membrane,

13 $$\begin{aligned} {\textbf {f}}^R_{ij}dt =\sqrt{2k_BT}\bigg (\sqrt{2\gamma ^T} d\overline{{\textbf {W}}^S_{ij}} + \sqrt{3\gamma ^C-\gamma ^T} \frac{tr[d{\textbf {W}}_{ij}]}{3} {\textbf {1}}\bigg )\cdot {\textbf {r}}_{ij}, \end{aligned}$$

where $d\overline{{\textbf {W}}^S_{ij}}=d{\textbf {W}}^S_{ij}-tr[d{\textbf {W}}^S_{ij} {\textbf {1}}/3]$ is the traceless symmetric part of a the Wiener increments matrix, the elements of which are taken from Normal distribution with mean 0 and standard deviation 1 [56]. The reason the random force is considered in addition to the dissipative force in the coarse-grained membrane model is that whenever a coarse-graining procedure is performed, dissipation and noise arise and both are related through a fluctuation–dissipation theorem [56]. These two additional force contributions were first introduced by Español in the context of the Fluid Particle Model [56].

The parameters of the membrane model are specified in Table 1 and taken from Reference [7], where a detailed description of the model and choice of its parameters can be found.

**Table 1:** Set of parameters used in the current work, taken from Reference [7]

Parameter	Simulation units value
Persistence length, *p*	0.00141
Viscosity parameter, $\gamma$	4
Cell area cons. constant, $k_A$	10,000
Cell volume cons. constant, $k_V$	15,000
Cell spring max length, $l_{max}^{cell}$	3
Cell spring equilibrium length, $l_{0}^{cell}$	0.5
Cell bending stiffness	65
Nucleus area cons. constant, $k_A$	5000
Nucleus volume cons. constant, $k_V$	15,000
Nucleus spring max length, $l_{max}^{nucl}$	1.2
Nucleus spring equilibrium length, $l_{0}^{nucl}$	0.5
Nucleus bending stiffness	250
Nuclear–cytoplasmic ratio	0.29

#### Cytoskeleton Model

The cytoskeleton is the cell component that typically contributes the most to the mechanics of the whole cell [1]. The cell cytoskeleton comprises a complex network of filaments, consisting mainly the actin filaments, microtubules, and intermediate filaments [58]. Here, we approximate the mechanical behavior of the cell cytoskeleton using a collection of viscoelastic Kelvin–Voigt (KV) bonds, linking cell and nucleus membrane particles. This system has been specifically selected because of the reduced number of parameters and its ability to capture the viscoelastic properties of the cytoskeleton in the numerical experiments described below. We have also explored a more general three-parameter standard linear solid (SLS) model [59], which produced results similar to those of the Kelvin–Voigt model. Given the comparable outcomes and the advantage of reduced complexity, we focus on the Kelvin–Voigt bond model in the main text, while the description of the SLS bond model and its corresponding results are provided in the Supplementary Material.

The Kelvin–Voigt bond can be represented as an elastic spring and a viscous damper placed in parallel and is accepted as a suitable model for simulating cellular behavior [60–63]. The parameters of a single KV bond are $k_{s}$ and $k_{v}$, that define the stiffness of the spring and the damper viscosity, respectively. Since the damper and the spring are connected in parallel, the strain of each component is identical,

14 $$\begin{aligned} \epsilon = \epsilon _{s} = \epsilon _{v}. \end{aligned}$$

The stress of the damper is defined as $\sigma _{v}=k_{v}\frac{\delta \epsilon _{v}}{\delta t}$, while the stress related to the spring is $\sigma _{s}=k_{s}\epsilon _{s}$. The total stress can therefore be expressed as the sum of the stresses of the spring and the damper,

15 $$\begin{aligned} \sigma _{tot}=k_{s} \epsilon (t)+k_{v} \frac{\delta \epsilon (t)}{\delta t}. \end{aligned}$$

We create the cytoskeleton structure by connecting the cell membrane particles to the nucleus membrane particles with multiple KV bonds. As a general procedure to reduce the computational complexity of the model and distribute the bonds uniformly across the cell, we employ k-means clustering [64] to restrict the set of membrane particles eligible for bonding. Additionally, different strategies can be employed for choosing pairs of particles to be connected by bonds, which may lead to different bond topologies and to distinct mechanical properties of cytoskeleton model. We demonstrate the impact of bond topology on the cell mechanics by defining two different models: Model 1 (M1) and Model 2 (M2). In Model 1, selected by k-means clustering eligible particles from the cell outer membrane are directly connected to the nearest eligible particles in the nucleus membrane. On the other hand, Model 2 connects each eligible particle in the outer membrane to the *w* closest eligible particles of the nucleus membrane. A 2D representation of the topologies considered for Model 1 and Model 2 are shown in Fig. 1c.

We keep membrane model parameters and in particular bond equilibrium length $l_0$ the same while modeling cells of different sizes, resulting in different number of membrane particles for these cells. Since the total number of bonds in the cytoskeleton model significantly affects the mechanics of the cell model, we introduce the concept of bond density, which is defined as a ratio of number of cytoskeleton bonds to the number of particles in the cell outer membrane,

$$\begin{aligned} \rho = \frac{\# \text {bonds in the cytoskeleton }}{ \# \text {particles in the outer membrane }}. \end{aligned}$$

Here, bond density $\rho$ is equal to the average number of bonds created for each particle in the outer membrane and is therefore independent of size of the modeled cell.

#### Nucleus

To model the nucleus, we treat it as a viscoelastic material and for simplicity, we employ the same construction as for the cytoskeleton with minor adjustments. A notable distinction arises from the necessity to connect particles within the same membrane; hence, we link the most distant particles rather than the closest ones. In addition, in order to reflect the fact that the nucleus was shown to be about 5 times stiffer than the surrounding cytoskeleton [65], we enforced the condition $k_s^{nuc} = 5 k_s^{cyt}$ , where $k_s^{nuc}$ and $k_s^{cyt}$ are the spring coefficients of the KV bonds in the nucleus and in the cytoskeleton, respectively. In contrast, the viscous coefficients are assumed to be the same $k_v^{nuc} = k_v^{cyt}$. From these assumptions it follows that the viscoelastic properties of the cytoskeleton and the nucleus in the model are mostly controlled by two parameters $k_s$ and $k_v$, with $k_s^{cyt} = k_s$, $k_s^{nuc} = 5 k_s$ , and $k_v^{cyt} = k_v^{nuc} = k_v$.

The parameters of the cytoskeleton and nucleus models will be calibrated using micropipette experimental data in Sect. 3.1.

#### Implementation

The cell model was implemented using LAMMPS (Large-scale Atomic/Molecular Massively Parallel Simulator) [66]. The simulations were carried out at Swiss National Supercomputing Centre (CSCS).

### Experimental Data

In order to calibrate and test performance of the proposed cell model, we utilize experimental data from Lykov et al. [7]. Below, we briefly summarize relevant experimental results.

#### Micropipette Aspiration

**Fig. 2:**
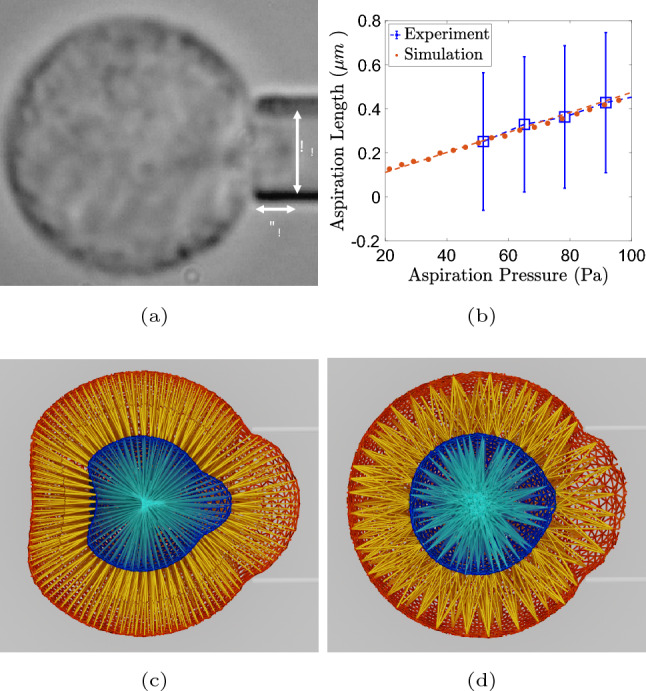
Micropipette aspiration experiments and simulations. **a** Microscopy image of the micropipette aspiration experiment (adapted from Reference [7]). **b** Normalized aspiration length as a function of aspiration pressure. **c** Visualization of the micropipette aspiration simulations employing M1 cell model. The back side of the cell model is pulled in the direction of the micropipette since it is linearly linked with the cell membrane particles aspired into the pipette. This behavior is also evident for the nucleus. **d** In M2 cell model simulations, this artifact is not present since cytoskeleton bond topology allows for more uniform distribution of stress inside the cell model.

The experimental approach employed for the assessment of cell elastic and viscous properties is the micropipette aspiration technique, utilizing a micropipette with a diameter of 4.25 $\mu$m and varying aspiration pressure (Fig. 2a). Specifically, the elasticity measurement involves the application of non-constant aspiration pressure, which progressively increased from 0 to 117.72 Pa at a constant rate of 3.27 Pa/s. The elasticity can be estimated by quantifying the extent of the cell aspired by the micropipette in relation to the aspiration pressure. To mitigate the influence of initial cellular configuration on outcomes, the normalized aspiration length $L_n$ is introduced: $L_n = (L_p - L_0/R_p)$, wherein $L_p$ represents the aspiration length, $L_0$ is the aspiration length under minute pressure, and $R_p$ denotes the radius of the pipette. Based on the obtained aspiration length dependence on aspiration pressure shown in Fig. 2b, it is also possible to get an estimate of the elastic modulus using Theret model [67], which is listed in Table 2. Cell viscous modulus, also listed in Table 2, is estimated utilizing data from the identical experiment, but using the evolution of aspiration length at constant aspiration pressure [7].

**Table 2:** Elastic and viscous moduli estimated from micropipette aspiration experimental data for MCF-10A cells used in this study

Elastic modulus	Viscous modulus
$237.47 \pm 66~\text {Pa}$	$10.2 \pm 3.5~\text {mPa}\cdot \text {s}$

#### Microfluidic Experiments

**Fig. 3:**
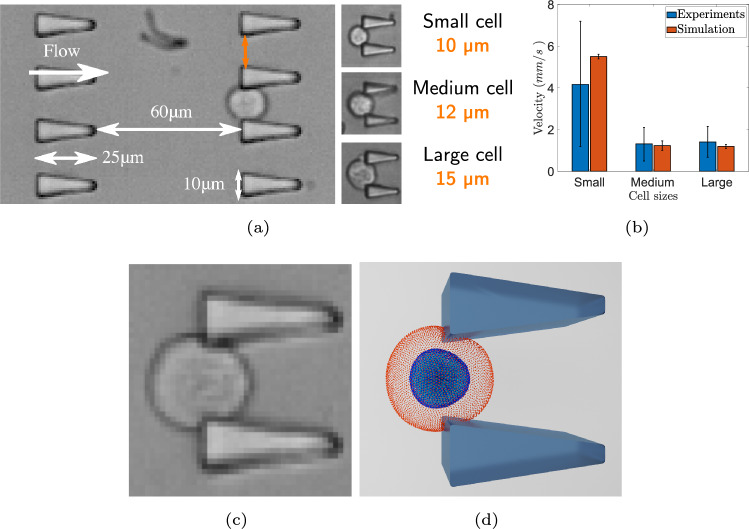
Microfluidic experiments and simulations. **a** Microscopy image of the microfluidic devices with different gap sizes used in experiments (adapted from Reference [7]). **b** Comparison of the transit velocities in microfluidic experiments and simulations for different cell sizes. **c** Snapshot of the MCF-10A cell passing through the obstacles in experiment (adapted from Reference [7]). **d** Visualization of the simulated cell passing through the obstacles.

The microfluidic devices in experiments consisted of a channel, 28 microns in height, with 10 rows of triangular-shaped obstacles (10 obstacles in each row, 60 $\mu$m between rows) as shown in Fig. 3a. The walls of microfluidic device were treated to ensure that there are no adhesive interactions of cells with the walls [7]. A prominent physical factor that plays a role in transit velocity of a cell passing through a constriction is its size. Therefore, cell population was divided into three categories of small (10–14 $\mu$m in diameter), medium (14–18 $\mu$m), and large (18–22 $\mu$m) cells. The distance between the obstacles in microfluidics devices was chosen to be 10, 12, and 15 microns for these three cell groups, respectively. The same pressure gradient driving the flow was used in all experiments. By adding small 3.85 $\mu$m beads to the flow in 12 $\mu$m gap size device, the average fluid velocity in the device was measured to be 22.57 mm/s. Experimental measurements of cell transit velocity for small, medium, and large cells are reported in Table 3. For a detailed description of the experiments we refer to Reference [7].

**Table 3:** Experimental data from the microfluidic experiment for each group of cells

Group	Cell size	Gap size	Cell velocity
Small	10–14 $\mu$m	10 $\mu$m	$4.16 \pm 3.03$ mm/s
Medium	14–18 $\mu$m	12 $\mu$m	$1.31 \pm 0.81$ mm/s
Large	18–22 $\mu$m	15 $\mu$m	$1.41 \pm 0.73$ mm/s

## Results

We first calibrated elastic and viscous properties of the proposed cell model using micropipette aspiration data, we then validated the calibrated model using data from the microfluidic experiment. Finally, we performed some parametric studies to quantify dependence of viscoelastic properties of cell model on its key parameters.

### Calibration: Micropipette Aspiration

In order to calibrate the model parameters, we first simulated a cell with a diameter of 16 $\mu$m, which represents the group of medium size cells used in experiments. Specifically, we focused on the two main parameters of the cytoskeleton model, $k_s$ and $k_v$, which, respectively, control elastic and viscous properties of the entire cell model. All other simulation parameters were taken from Reference [7] and are reported in Table 1.

By testing both cell models M1 and M2 in micropipette aspiration simulations, we noticed that the M1 cell model contains chains of bonds which linearly connect the two opposite sides of the cell. This structural feature produces an artifact in the micropipette aspiration simulations, where the stress applied on one side of the cell directly propagates to the other side, resulting in a concave cell geometry opposite to the micropipette, which violates the principle of local action, as can be seen in Fig. 2c. This artifact is absent in micropipette aspiration simulations of M2 cell model with $w=5$ (Fig. 2d), in which the cytoskeleton bond topology allows for more uniform distribution of stress inside the cell model. The value of $w = 5$ was empirically identified as the minimum necessary to eliminate the artifact. Given these results, we decided to use only the second cell model, namely, M2 with $w=5$, in all studies described below.

We found the value of stiffness parameter $k_s$ by matching experimental data of cell aspiration length as a function of aspiration pressure in simulations, as shown in Fig. 2b. With the obtained value of $k_s = 120$, the estimated value of the elastic modulus based on the Theret model [67] is 230 Pa, which is close to the value reported for the MCF-10A cells in Table 2. Viscous parameter $k_v$ was then estimated using similar micropipette simulations but under constant aspiration pressure. We matched the experimental estimate of cell viscous modulus of 11.11 mPa$\cdot$s reported in Table 2, by setting the value of $k_v$=10 and fitting the Theret Model [68]. With both parameters $k_s$ and $k_v$ defined, models of small and large cells, 12 $\mu$m and 20 $\mu$m in diameter, were created by keeping the same cytoskeleton bond density $\rho = 0.8$, ensuring size-invariant mechanical properties of the models.

The parameters of the cytoskeleton and nucleus models obtained after calibration are summarized in Table 4.

**Table 4:** Parameters of the cytoskeleton and nucleus models used in this study

Parameter	Simulation units value
stiffness cytoskeleton, $k_s^{cyt}$	120
stiffness nucleus, $k_s^{nucl}$	600
viscosity cytoskeleton, $k_v^{cyt}$	10
viscosity nucleus, $k_v^{nucl}$	10
density bonds cytoskeleton, $\rho ^{cyt}$	0.8
density bonds nucleus, $\rho ^{nucl}$	0.8
bond topology, *w*	5

### Validation: Microfluidic Simulations

Having established the parameters for cell stiffness and viscosity, we continued with the validation of the cell model using microfluidic experiment data, following the methodology employed in [7].

The solid walls of microfluidic device in simulations were assembled from randomly distributed DPD particles whose positions were fixed. In addition, bounce-back reflections were used to achieve no-slip conditions and prevent fluid particles from penetrating the walls [69, 70]. The cell model was immersed into the DPD fluid. Its particles interacted with fluid particles through the DPD forces, so that the cell motion was fully coupled with the surrounding fluid motion. The magnitude of the body force driving the flow was established by matching the average fluid velocity of 22.57 mm/s in 12 $\mu$m gap size device. The same body force was then used in all simulations. Comparison of average transit velocity in simulations and experiments for small, medium, and large cells is shown in Fig. 3b. In Fig. 3c and d, snapshots from experiment and simulations illustrating deformation of medium size cell are shown. The viscous response of the cells was quantified by measuring the relaxation time, defined as the time required for a cell to recover its original shape after deformation. The ratio of the relaxation time to total transit time was then compared to experimental measurements and is reported for small, medium, and large cells in Table 5. In general, we find the simulation results to be in a good agreement with experimental measurements.

**Table 5:** The ratio of the relaxation time to total transit time in the microfluidic experiment [7] and the present study

Cell model	Experiment	Simulation
Small Cell	0.35	0.35 ± 0.04
Medium Cell	0.109	0.13 ± 0.05
Large Cell	0.119	0.22 ± 0.02

### Effects of Cytoskeleton Model Parameter Variation

**Fig. 4:**
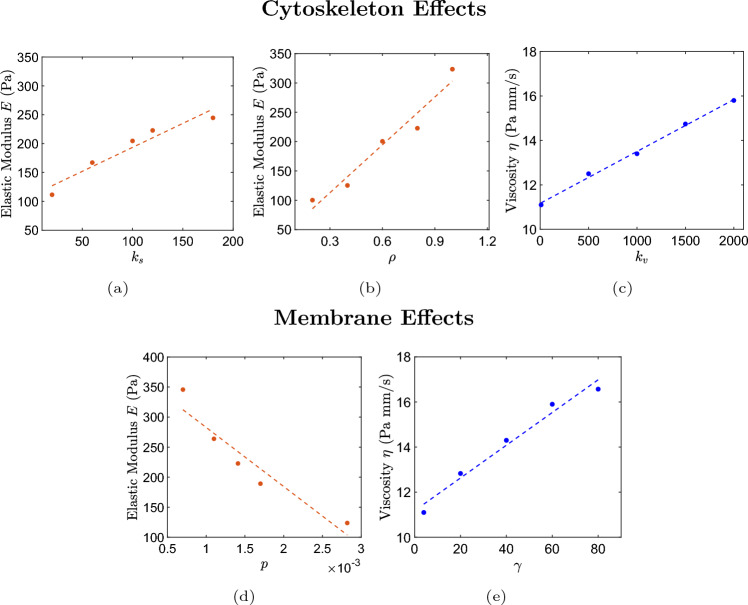
Effect of variation of cytoskeleton and membrane model parameters on the entire cell model properties. Cytoskeleton effects: **a**, **b** results for the effect of the cytoskeleton KV bond stiffness coefficient $k_s$ and density $\rho$ variation on cell elastic modulus, also known as Young’s modulus, estimated based on Theret model [67] from micropipette aspiration simulations. **c** Results for the effect of the cytoskeleton KV bond viscous coefficient $k_v$ variation on the cell viscous modulus estimated based on Theret model [68] from micropipette aspiration simulations. Membrane effects: **d** results for the effect of membrane persistence length parameter *p* variation on cell elastic modulus, estimated based on Theret model [67] from micropipette aspiration simulations. **e** Results for the effect of the membrane dissipative force parameter $\gamma$ variation on the cell viscous modulus, estimated based on Theret model [68] from micropipette aspiration simulations.

The viscoelastic properties of the cell model are defined by the values of its parameters. At small and moderate deformations, parameters of the cytoskeleton model and membrane model primarily define the properties of the entire cell, while at large deformations the properties of the nucleus model may become important as well [7]. We consider here the effect of variation of the new cytoskeleton model parameters. In order to measure cell model properties, we utilize the micropipette aspiration simulations, providing estimates based on the Theret model [67, 68], as often done in experiment. The stiffness coefficient $k_s$ of the KV bonds and bond density $\rho$ in the cytoskeleton model control elastic properties of the cell model. Cell stiffness can be varied independently by changing $k_s$ or $\rho$ as shown in Fig. 4a, b. Cell viscous properties can be adjusted by changing viscous coefficient $k_v$ of the KV bonds (Fig. 4c).

### Effects of Membrane Model Parameter Variation

The dependence of the model’s viscoelastic properties on its membrane parameters was extensively studied in Reference [7]. In this work, we investigate the effect of persistence length (*p*) and membrane viscosity ($\gamma$). Specifically, we observe an inverse relationship between persistence length and cell elastic modulus, with the modulus decreasing linearly as persistence length increases, as illustrated in Fig. 4d. Additionally, by adjusting the membrane viscosity using parameter $\gamma$, we can directly influence the overall viscosity of the cell ($\eta$), as shown in Fig. 4e.

These results, along with those obtained in the previous section, highlight that without additional experimental data, defining a unique set of model parameters is not possible. Consequently, various parameter combinations can yield the same mechanical properties for the entire cell model.

### Computational Efficiency

**Fig. 5:**
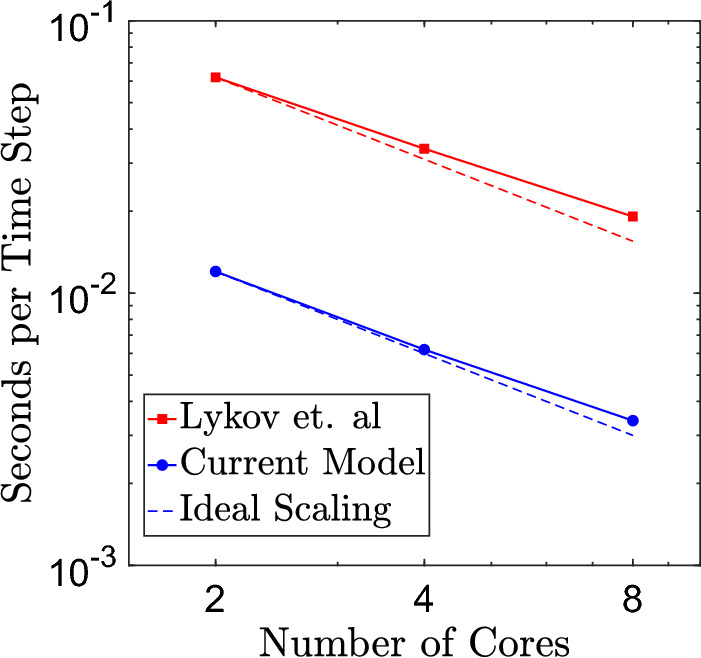
Comparison of scaling behavior and execution time per simulation time step between the large size cell model by Lykov et al. [7] and the model developed in the present study.

Apart from the particles used for cell and nucleus membranes discretization, the proposed cell model does not introduce any additional particles. The membrane model, which was originally developed for modeling red blood cells [16–18], allows efficient implementation with flow simulations including up to 1.43 Billion RBCs [40]. The model of Lykov et al. [7] includes a detailed description of the cytoskeleton structure in addition to the membranes, requiring approximately 47, 000 particles for simulating a cell with a diameter of 20 $\mu$m. In contrast, simulations of the same cell in the present study required only 7, 212 particles, significantly reducing computational effort and providing a speedup of 4.7x. We list in Table 6 the total number of particles, bonds, angles, and dihedrals required for implementation of both models in LAMMPS. A comparison of the scaling behavior and execution time per time step for the two models is presented in Fig. 5. For simulations in which remodeling of the cytoskeleton does not play significant role or can be omitted based on other considerations, the proposed model can be used as rather inexpensive alternative, while still capturing the viscoelastic properties of the eukaryotic cell within a range of deformations considered here.

**Table 6:** Comparison of total number of particles, bonds, angles, and dihedrals required for large size cell model implementation in LAMMPS for Lykov et al. [7] and the present study

Cell model	Particles	Bonds	Angles	Dihedrals
Lykov et al.[7]	47,050	39,539	42,474	25,294
Current model	7212	27,415	14,416	21,624

## Discussion

In this study, we presented a simplified yet robust computational model for simulating the mechanical properties of eukaryotic cells, specifically focusing on the cell membrane, nucleus, and cytoskeleton. By reducing the complexity of the cytoskeleton model employed in previous studies [7] through the use of viscoelastic Kelvin–Voigt bonds, we significantly decreased the computational load while retaining the ability to capture the viscoelastic behavior of cells. Our results demonstrated that the bond topology within the cytoskeleton model significantly influences the accuracy of the simulations, leading us to avoid a naive distribution of bonds to ensure realistic stress distribution and cell deformation behavior. The resulting cell model was calibrated and validated using experimental data from micropipette aspiration and microfluidic experiments involving breast epithelial cells (MCF-10A). While the simplifications introduced in the model reduced the number of parameters and computational complexity, they did not compromise the model’s ability to produce reliable results. We believe this balance between simplicity and accuracy makes the proposed model a valuable tool for simulating eukaryotic cell mechanics in flow, enabling faster simulations, while also simplifying parameterization procedure. Additionally, integrating the model with machine learning techniques could further enhance its predictive capabilities and facilitate the automated discovery of optimal model parameters, as demonstrated in recent studies [71].

## Supplementary Information

Below is the link to the electronic supplementary material.

(PDF 283 kb)

## Data Availability

There are no datasets for the current study.
