# Supplementary material for: A Computationally Efficient Viscoelastic Eukaryotic Cell Model
Source: Ann Biomed Eng. 2025 Jun 19;53(9):2047–58. doi: 10.1007/s10439-025-03772-5 (PMC12391228; doi:10.1007/s10439-025-03772-5)
Supplement: Supplementary file 1 — (PDF 283 kb) [file 10439_2025_3772_MOESM1_ESM.pdf]

Annals of Biomedical Engineering

Supplementary material for: A computationally efficient viscoelastic eukaryotic cell model

Pietro Miotti[1,2†], Matteo Scarpone[1†], Chwee Teck Lim[3,4,5], Igor V. Pivkin[1,2*]

[1*]Institute of Computing, Faculty of Informatics, Università della Svizzera italiana, Lugano, Switzerland.
[2]Swiss Institute of Bioinformatics, Lausanne, Switzerland.
[3]Institute for Health Innovation and Technology (iHealthtech), National University of Singapore, Singapore, Singapore.
[4]Department of Biomedical Engineering, National University of Singapore, Singapore, Singapore.
[5]Mechanobiology Institute, National University of Singapore, Singapore, Singapore.

*Corresponding author(s). E-mail(s): igor.pivkin@usi.ch;
Contributing authors: pietro.miotti@usi.ch; matteo.scarpone@usi.ch; ctlim@nus.edu.sg;
†These authors contributed equally to this work.

Three-Parameter Standard Linear Solid Model

In addition to the Kelvin-Voigt (KV) bond model described in the main text, we implemented a more general three-parameter standard linear solid (SLS) model. Represented in its Maxwell form, the SLS model consists of two parallel systems: a Maxwell element, comprising a spring with elastic modulus k_{s1} and a dashpot with viscosity k_v arranged in series, coupled with an elastic element with elastic modulus k_{s2} [1]. The governing differential equation, derived from the constitutive relationships, is given by

$$\sigma(t) + \frac{k_v}{k_{s2}} \frac{d\sigma(t)}{dt} = k_{s1}\varepsilon(t) + k_v \frac{k_{s1} + k_{s2}}{k_{s2}} \frac{d\varepsilon(t)}{dt}.$$

We evaluated the performance of the SLS model by replacing the KV bonds in both the cytoskeleton and nucleus models. The parameters for the cytoskeleton and nucleus models with SLS bonds were calibrated using micropipette experimental data, as described in Section 3.1 of the main text. The obtained parameters are summarized in Table S1. A comparison of normalized aspiration length as a function of aspiration pressure shows good agreement between the SLS and KV model results after parameter calibration (Fig. S1). Similar consistency was observed in the microfluidic experiment simulations (Fig. S2).

Table S1: Set of parameters of the cytoskeleton used for the SLS model

Parameter	Simulation units value
stiffness cytoskeleton, $k_{s1}^{cyt} = k_{s2}^{cyt}$	120
stiffness nucleus, $k_{s1}^{nucl} = k_{s2}^{nucl}$	600
viscosity cytoskeleton, k_v^{cyt}	10
viscosity nucleus, k_v^{nucl}	10
density bonds cytoskeleton, ρ^{cyt}	0.8
density bonds nucleus, ρ^{nucl}	0.8
bond topology, w	5

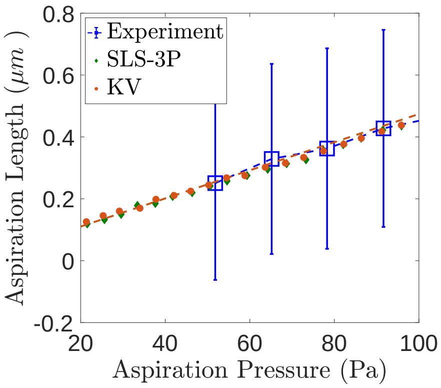

Fig. S1: Comparison of normalized aspiration length as a function of aspiration pressure in micropipette experiments and simulations with SLS and KV bond models for medium size cells.

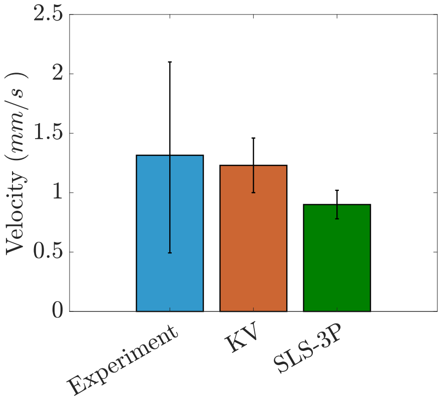

Fig. S2: Comparison of the transit velocities in microfluidic experiments and simulations with SLS and KV bond models for medium size cells.

References

[1] Fung, Y.-C.: Biomechanics, Mechanical Properties of Living Tissues, (1993). https://doi.org/10.1007/978-1-4757-2257-4
